# Treatment of Progressive Multifocal Leukoencephalopathy With Third-Party Allogeneic BK Virus T Cells

**DOI:** 10.1093/cid/ciag404

**Published:** 2026-07-06

**Authors:** Amanda Olson, Ye Li, David Marin, Peter F Thall, Roland L Bassett, Melissa Barnett, Rafet Basar, Pinaki P Banerjee, T Anne Kleiman, Merry Chen, Jennie Rexer, Max Wintermark, Jeanie Choi, Kim Learned, Indreshpal Kaur, Melihate Sylejmani, Glorette Abueg, Roy F Chemaly, Victor Mulanovich, Rejeena Shrestha, Nadima Uprety, Karla M Castro, May Daher, Isabel M Galvan, Dominique Washington, Richard E Champlin, Elizabeth J Shpall, Katayoun Rezvani

**Affiliations:** Department of Stem Cell Transplantation and Cellular Therapy, The University of Texas MD Anderson Cancer Center, Houston, Texas, USA; Department of Stem Cell Transplantation and Cellular Therapy, The University of Texas MD Anderson Cancer Center, Houston, Texas, USA; Institute for Cell Therapy Discovery and Innovation, The University of Texas MD Anderson Cancer Center, Houston, Texas, USA; Department of Stem Cell Transplantation and Cellular Therapy, The University of Texas MD Anderson Cancer Center, Houston, Texas, USA; Department of Biostatistics, The University of Texas MD Anderson Cancer Center, Houston, Texas, USA; Department of Biostatistics, The University of Texas MD Anderson Cancer Center, Houston, Texas, USA; Department of Stem Cell Transplantation and Cellular Therapy, The University of Texas MD Anderson Cancer Center, Houston, Texas, USA; Department of Stem Cell Transplantation and Cellular Therapy, The University of Texas MD Anderson Cancer Center, Houston, Texas, USA; Institute for Cell Therapy Discovery and Innovation, The University of Texas MD Anderson Cancer Center, Houston, Texas, USA; Department of Stem Cell Transplantation and Cellular Therapy, The University of Texas MD Anderson Cancer Center, Houston, Texas, USA; Institute for Cell Therapy Discovery and Innovation, The University of Texas MD Anderson Cancer Center, Houston, Texas, USA; Department of Neuro-Oncology, The University of Texas MD Anderson Cancer Center, Houston, Texas, USA; Department of Neuro-Oncology, The University of Texas MD Anderson Cancer Center, Houston, Texas, USA; Section of Neuropsychology, Department of Neuro-Oncology, The University of Texas MD Anderson Cancer Center, Houston, Texas, USA; Department of Radiology, The University of Texas MD Anderson Cancer Center, Houston, Texas, USA; Department of Radiology, The University of Texas MD Anderson Cancer Center, Houston, Texas, USA; Department of Radiology, The University of Texas MD Anderson Cancer Center, Houston, Texas, USA; Department of Stem Cell Transplantation and Cellular Therapy, The University of Texas MD Anderson Cancer Center, Houston, Texas, USA; Department of Stem Cell Transplantation and Cellular Therapy, The University of Texas MD Anderson Cancer Center, Houston, Texas, USA; Department of Stem Cell Transplantation and Cellular Therapy, The University of Texas MD Anderson Cancer Center, Houston, Texas, USA; Department of Infectious Diseases, Infection Control, and Employee Health, The University of Texas MD Anderson Cancer Center, Houston, Texas, USA; Department of Infectious Diseases, Infection Control, and Employee Health, The University of Texas MD Anderson Cancer Center, Houston, Texas, USA; Department of Stem Cell Transplantation and Cellular Therapy, The University of Texas MD Anderson Cancer Center, Houston, Texas, USA; Institute for Cell Therapy Discovery and Innovation, The University of Texas MD Anderson Cancer Center, Houston, Texas, USA; Department of Stem Cell Transplantation and Cellular Therapy, The University of Texas MD Anderson Cancer Center, Houston, Texas, USA; Institute for Cell Therapy Discovery and Innovation, The University of Texas MD Anderson Cancer Center, Houston, Texas, USA; Department of Stem Cell Transplantation and Cellular Therapy, The University of Texas MD Anderson Cancer Center, Houston, Texas, USA; Department of Stem Cell Transplantation and Cellular Therapy, The University of Texas MD Anderson Cancer Center, Houston, Texas, USA; Institute for Cell Therapy Discovery and Innovation, The University of Texas MD Anderson Cancer Center, Houston, Texas, USA; Department of Stem Cell Transplantation and Cellular Therapy, The University of Texas MD Anderson Cancer Center, Houston, Texas, USA; Department of Stem Cell Transplantation and Cellular Therapy, The University of Texas MD Anderson Cancer Center, Houston, Texas, USA; Department of Stem Cell Transplantation and Cellular Therapy, The University of Texas MD Anderson Cancer Center, Houston, Texas, USA; Department of Stem Cell Transplantation and Cellular Therapy, The University of Texas MD Anderson Cancer Center, Houston, Texas, USA; Department of Stem Cell Transplantation and Cellular Therapy, The University of Texas MD Anderson Cancer Center, Houston, Texas, USA; Institute for Cell Therapy Discovery and Innovation, The University of Texas MD Anderson Cancer Center, Houston, Texas, USA

**Keywords:** progressive multifocal leukoencephalopathy (PML), JC polyomavirus (JCV), BK virus-specific T cells (BK-VSTs), adoptive T-cell therapy, immunotherapy

## Abstract

**Background:**

Progressive multifocal leukoencephalopathy (PML) is a fatal demyelinating disease caused by JC polyomavirus that affects immunosuppressed individuals and has no approved therapy. Here, we evaluate the safety and efficacy of third-party, off-the-shelf, partially HLA-matched BK virus-specific T cells (BK-VST) in patients with PML.

**Methods:**

We conducted a single-center phase 2 study (including 3 patients treated under compassionate or emergency, INDs); 37 patients received most closely HLA-matched BK-VST at a median dose of 2.0 × 10^5^ T cells/kg, with additional infusions permitted for subjects who did not achieve a complete response (CR). Twenty-three patients had an underlying diagnosis of hematologic malignancy. Each patient received a median of 2 doses (range, 1–12; IQR 2). Overall response (OR) was defined as virologic response with neurologic stabilization or improvement.

**Results:**

During 12 months of follow-up, 21 patients (56.8%) achieved an OR, including 16 (43.2%) with a CR or CR with neurologic sequelae (CRnesq). Responses were rapid (median 23 days) and durable, with no loss of response observed despite limited HLA matching. The 1-year overall survival was 61.1% (95% CI, 41.9–77.4). Patients achieving an OR after the first infusion had markedly improved 1-year survival compared with nonresponders (93.3% vs 0%; *P* < .001). Greater HLA matching correlated with clinical benefit.

**Conclusions:**

These data demonstrate that third-party BK-VST infusions are safe and potentially effective, supporting a scalable immunotherapeutic approach for this otherwise fatal disease.

**Clinical Trials Registration.** NCT02479698.

## Teaser

Off-the-shelf, partially HLA-matched BK virus-specific T cells demonstrate safety and efficacy in PML, achieving 56.8% overall response, rapid durable viral clearance, and 61.1% 1-year survival, supporting a scalable immunotherapy for this otherwise fatal disease.

Progressive multifocal leukoencephalopathy (PML) is a rare but often fatal infection of the central nervous system caused by JC polyomavirus (JCV), for which no established treatment exists [1, 2]. Up to 70% of healthy adults harbor latent, asymptomatic JCV after primary infection. Reactivation in immunosuppressed individuals causes demyelination, resulting in altered mentation, motor and visuospatial deficits, ataxia, and death in up to 50%–80% of cases [3–8]. PML has been associated with a range of immunocompromised states, including malignancy, transplantation, AIDS, and treatment with immunosuppressive agents [3–5, 9]. Outcomes vary by underlying cause, with malignancy-associated PML, particularly those with hematologic malignancy, carrying the poorest prognosis [10, 11]. In rare cases, immune reconstitution may clear the virus [9]. Attempts to treat PML with antivirals and PD-1 inhibition have yielded mixed success [12, 13].

JCV and BK virus (BKV) are members of the *Polyomaviridae* family and share immunodominant antigens, including large and small tumor antigens, and the VP1, VP2, and VP3 capsid proteins [14–16]. Third-party polyomavirus-specific cytotoxic T-lymphocytes have shown safety and efficacy in treating JCV-associated PML [10, 17–21]. Virus-specific T cells (VSTs) may be generated using several methods including direct cell selection, rapid expansion, and classic ex vivo expansion [22]. In this protocol, VSTs were generated using a rapid expansion method as described in detail in Supplemental Appendix. Herein, we report results from a clinical trial using third-party, cryopreserved, most closely HLA-matched BK-VSTs in 37 immunosuppressed patients with PML.

## METHODS

### Clinical Trial Design

This single-center, Phase 2 study (NCT02479698) was conducted at MD Anderson Cancer Center to evaluate the efficacy of adoptive therapy with BK-VST cells in patients with symptomatic BKV and JCV infection [23]. This report focuses on an interim analysis of the PML-only subgroup, undertaken to provide an early assessment of efficacy in this high-risk population. All patients or their power of attorney provided written informed consent. BK-VSTs were generated from healthy volunteer donors and cryopreserved.

### Patients

From April 2016 to May 2023, 37 patients with PML aged 7–83 (median 61) received off-the-shelf, partially HLA-matched BK-VSTs. All met American Academy of Neurology diagnostic criteria for PML with JCV DNA detected in CSF (n = 34) or by histopathology confirmation from brain biopsies (n = 3) [24]. Early in the trial, three patients were treated under a Compassionate IND (CIND) or Emergency IND (EIND). The trial exclusion criteria were later revised, making all patients eligible for treatment under the current protocol version. These amendments addressed initial challenges, such as the exclusion of patients without malignancy or those with AIDS. The changes also increased the number of infusions allowed. A separate Institutional Review Board (IRB)-approved protocol was used to retrospectively analyze the data for patients treated under CIND/EIND. All authors affirm the integrity of the data and protocol compliance. Patients with a prior diagnosis of PML were eligible if they demonstrated new or progressive neurological symptoms or MRI findings consistent with progressive disease. This reflects the rationale that PML may remain active or re-emerge under ongoing immunosuppression, and treatment was directed toward patients with evidence of active disease progression rather than quiescent or resolved infection. The underlying causes of immunosuppression are detailed in the Supplementary Methods.

Because most patients were referred from outside institutions and symptom onset data were often unreliable or unavailable, time from referral to treatment was used as a surrogate for disease timeline. Accrual occurred over several years reflecting the rarity of PML, the complexity of early diagnosis and the need for interinstitutional referral after diagnostic confirmation.

### Generation of BK-VSTs

BK-VSTs were generated from healthy donor PBMCs by stimulation with overlapping peptides from 5 immunodominant polyomavirus antigens. Manufacturing and quality testing are described in Supplementary Methods.

### Characterization of BK-VSTs

Patients received biobanked VST cell lines generated from 38 healthy donors, comprising CD4+ and CD8+ T cells (Supplementary Figure 1A). Functional characterization is shown in Supplementary Figure 1B–D.

### Selection of VSTs for Clinical Use

At MD Anderson Cancer Center, patients were assigned the best available partially HLA-matched VST line from an established bank. The VST with the highest match based on matching at HLA-A, -B, and -DRB1 was selected for infusion at a median dose of 2 × 10^5^ T cells/kg.

### Clinical and Virologic Endpoints

Complete response (CR) was defined as the absence of neurologic dysfunction and undetectable JCV in CSF by qPCR (Viracor Laboratories, Lancaster, PA; limit of detection <72 copies/mL).

Complete virologic response with persistent neurologic sequelae (CRnesq) was defined as clinical improvement with undetectable JCV in CSF. Given the potential for irreversible neurologic damage despite viral clearance, CR and CRnesq were combined for analysis. Of note, this designation does not necessarily indicate complete neurologic recovery.

Virologic response (VR) was defined as >50% decrease in CSF JCV viral load without clinical or MRI progression, based on previously published criteria.

Overall response (OR) included CR, CRnesq, or VR.

Nonresponse or progression was defined as new neurologic symptoms or increased viral load.

### Imaging Endpoints

All patients underwent baseline MRI prior to BK-VST administration and at serial intervals post-treatment. Imaging was performed on 3T MRI scanners (GE and Siemens Healthcare), using standard T1, T2 diffusion-weighted imaging, fluid-attenuated inversion recovery (FLAIR), gradient echo, and postcontrast T1-weighted sequences. All scans were independently reviewed by 3 experienced neuroradiologists blinded to clinical data. Qualitative assessments included changes in white matter signal abnormalities (categorized as improved, stable, or worsened), the presence of diffusion restriction, contrast enhancement or other radiographic features consistent with an inflammatory response, age-appropriate brain volume, and involvement of the cerebellum or brainstem. The assessment time points are included in Supplementary Table 1.

### Assessment of BK-VST Response Following Adoptive Infusion by CyTOF

Mass cytometry was performed as previously described [25]. Antibodies are listed in Supplementary Table 2A and B. For intracellular staining, cells were permeabilized using 80% methanol, stained with intracellular marker-specific antibodies, followed by Iridium nucleic acid intercalator (Standard BioTools). Cells were acquired on a Helios instrument (Standard BioTools) and data analyzed by Cytobank.

### Statistical Methods

Statistical analyses were primarily descriptive, reflecting the exploratory nature and limited sample size of the study. Overall survival (OS) was estimated using the Kaplan–Meier method [26], with all patients censored at their last known follow-up date if they had not reached 1 year. The cumulative incidence of response was calculated using a competing risks framework, with death as the competing event. Further statistical details, including prior specifications, model assumptions, and computational settings are provided in the Supplementary Methods.

## RESULTS

### Infusion of BK-VSTs Induces Sustained Clinical Responses With MRI Improvements in Patients With PML

Thirty-seven consecutive patients with PML received off-the-shelf BK-VSTs at our center (Supplementary Figure 2, CONSORT flow diagram). Patients received a median of 2 infusions (range 1–12; IQR 2) at monthly intervals until achieving CR or clinical deterioration. The number of infusions varied according to individual clinical response, underlying disease, degree of immunosuppression and treating physician assessment. Patients achieving CR required fewer infusions, while those with persistent disease received additional doses. Thirty-five patients (95%) received their first VST infusion within 4 months of PML diagnosis (median 0.9 months, range 0.1–3.9; IQR 1.2). Two additional patients received their first infusion at 48.0 and 54.3 months post-diagnosis (see below). The median BK-VST dose was 2.0 × 10^5^ cells/kg (range 1.0–2.0 × 10^5^; IQR 0). VST lines were matched to recipients at a minimum of 1/6 HLA alleles (median 3, range 1–5; IQR 2).

The median follow-up for surviving patients was 228 days (range 5–365 days; IQR 309). Eleven patients died during the study. The estimated 1-year probability of OS was 61.1% (95% CI 41.9–77.4). During the 12-month study follow-up, 21 patients (56.8%) achieved an OR, including 16 (43.2%) with a CR (n = 5) or CR with neurological sequelae (CRnesq) (n = 11), which were grouped for analysis. An additional 5 patients (13.5%) achieved a VR without clinical improvement. Responses were rapid, with a median time to OR of 23 days (range 6–32; IQR 8.5). The 12-month cumulative incidence of CR/CRnesq was 62.6% (95% CI 40.1%–78.5%) (Figure 1). Importantly, no patient lost a previously achieved response during follow-up.

**Figure 1.**
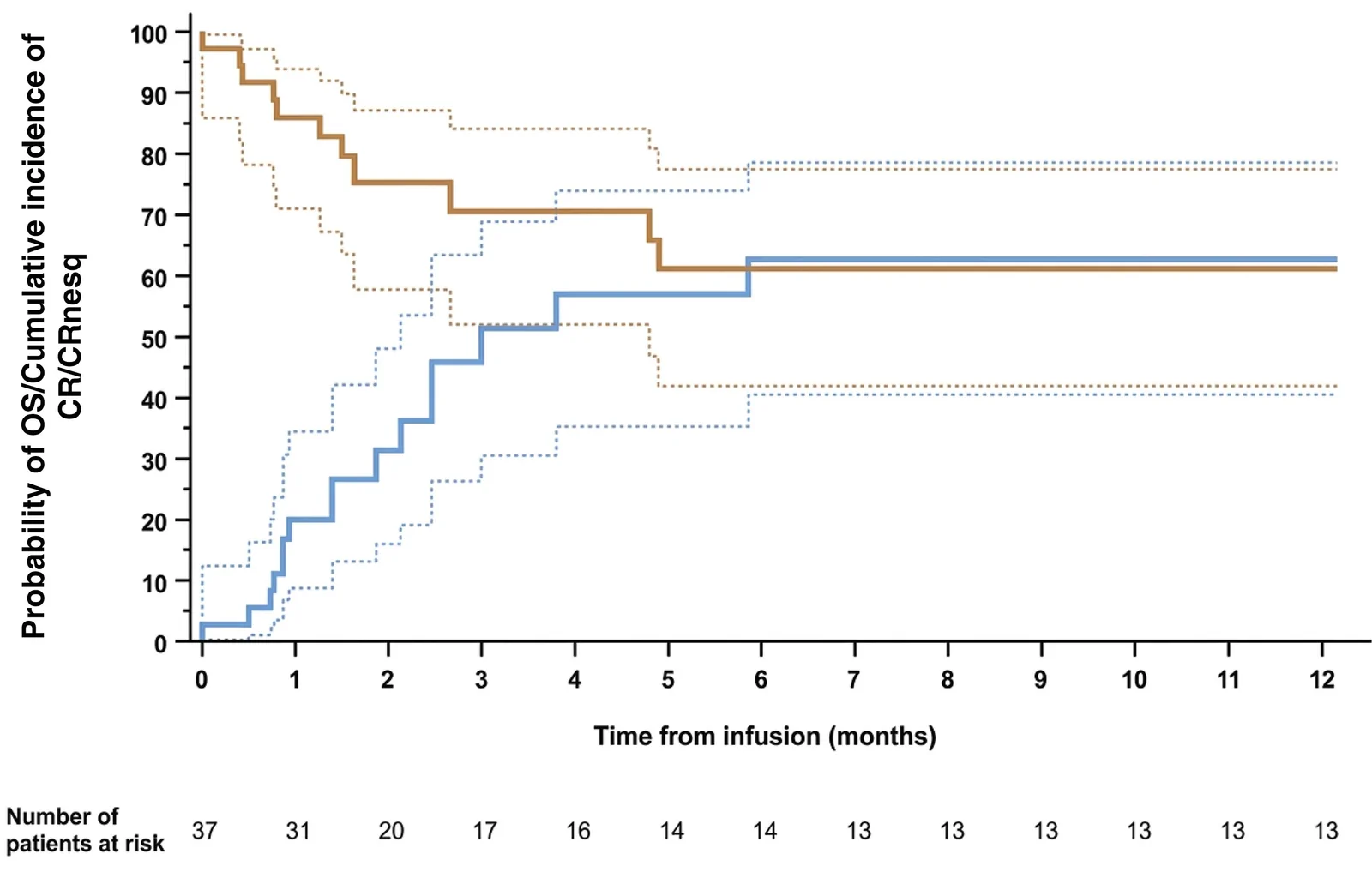
Patient outcomes. One-year probability of overall survival (brown) and 1-year cumulative incidence of CR/CRnesq (blue). Dotted lines represent the upper and lower margins of the 95% CIs. The 1-year probability of OS was 61.1% (95% CI 41.9%–77.4%) and the 1-year cumulative incidence of CR/CRnesq was 62.6% (95% CI 40.1%–78.5%). Abbreviations: CR, complete response; CRnesq, complete virologic response with persistent neurologic sequelae; OR, overall response.

The Modified Rankin Scale (mRS), which rates disability from 0 (no symptoms) to 6 (death) [27] was assessed by 2 study neurologists. Among the 11 patients with CRnesq, median mRS improved from 4 (range 2–4; IQR 1), indicating moderately severe disability, to 2 (range 1–3; IQR 2), reflecting slight disability at last follow-up (Supplementary Table 3).

Cognitive outcome was assessed in 22 patients at baseline (T1). Of these, 10 returned for post-treatment evaluation (T2; median 116.5 days postbaseline), 4 for a third assessment (T3; median 154.5 days post-T2), and 2 for a fourth assessment (T4; 1568 and 251 days post-T3) (Supplementary Table 4). Learning and memory performance was summarized using composite z-scores derived from the Hopkins Verbal Learning Test-Revised (n = 9) or Brief Visuospatial Memory Test-Revised (n = 1, due to expressive aphasia). Paired samples *t*-tests assessed group-level comparisons between T1 and T2, and the reliable change index (RCI) was applied to identify within-person changes [28–31].

At the group level, composite scores improved from a mean z-score of −2.35 (SD 1.53) to −1.85 (SD 1.49), although the change did not reach significance (*t*(9) = −1.70, *P* = .123), despite a moderate effect size (Cohen's *d* = 0.54), likely reflecting underpowering due to the small sample size (n = 10). However, at the individual level, RCI analysis indicated that 80% of patients demonstrated improvement between T1 and T2. However, most remained within the impaired range compared with demographically matched normative data (Supplementary Figure 3).

MRI findings showed that 13 of 15 imaged patients with CR/CRnesq (87%) had a reduction in the extent of FLAIR lesions over time, while the remaining 2 (13%) had stable FLAIR lesion burden. Notably, none of the responders experienced radiographic progression. In contrast, among nonresponders, FLAIR lesion progression was observed in 15 patients (79%), with 4 (21%) remaining stable and none exhibiting radiographic improvement. Two nonresponders and one responder did not have follow-up MRIs. Diffusion restriction was more common in nonresponders (26%) than in responders (13%). Radiographic features consistent with an inflammatory response were observed in 73% of responders versus 26% of nonresponders. Notably, the majority of these patients did not develop clinical symptoms of IRIS. Brain atrophy was more common in responders with neurologic sequelae (40%) than those without (20%). The anatomical distribution of FLAIR lesions did not differ between responders and nonresponders.

Concomitant pembrolizumab treatment was permitted at the treating physician's discretion for patients who did not achieve at least a partial response after the first VST infusion. However, some patients with progressive disease were not eligible for pembrolizumab because of their underlying condition, including solid organ transplant recipients. Seven patients received pembrolizumab in combination with BK-VSTs. Six began treatment shortly after their first VST infusion, with 4 receiving a single dose and 2 receiving 2 doses of pembrolizumab (median 13.5 days post-VST infusion; range: 4-29 days). Among them, 2 achieved VR, including one deepening to CR. The remaining 4 showed no response. A seventh patient, who had already achieved VR with VSTs, started pembrolizumab on day +105 and received 5 monthly doses, ultimately achieving CR. No toxicity was observed with the combination of pembrolizumab and BK-VSTs.

We compared outcomes across 3 groups: (1) 28 patients who did not receive pembrolizumab and were treated exclusively with VSTs alone (within 4 months of diagnosis); (2) 9 patients who either received BK-VSTs plus pembrolizumab (n = 7) or had a prolonged disease course (n = 2), and (3) the entire trial cohort. The 2 patients with prolonged disease were included in the second group because their extended interval between PML diagnosis and VST infusion created a distinct, nonstandard clinical course with potential survivor bias. This allowed the primary VST monotherapy analysis to remain focused on patients treated early after diagnosis, in whom outcomes could be more directly attributed to VST treatment. Outcomes were similar across the 3 groups (Supplementary Table 5), supporting early VST monotherapy as an effective treatment strategy and indicating that pembrolizumab can be incorporated in selected cases without added toxicity.

### Achievement of OR at Day 28 After the First Infusion of BK-VSTs is Strongly Predictive for Superior Overall Survival

Thirty one of 37 patients were evaluable for response assessment after the first BK-VST infusion. Six patients were not evaluable as 4 patients died from progressive PML within the first month on days +12, +13, +23, and +24 post-VST infusion and 2 withdrew from the study without response on days +5 and +26 and were lost to follow-up. Among the 31 evaluable patients, 21 achieved an OR by day 28 after the first BK-VST infusion. These responders had markedly superior 1-year OS than nonresponders (93.3% vs 0%, *P* < .001) (Figure 2). One patient who initially achieved CR later died from complications of underlying leukemia.

**Figure 2.**
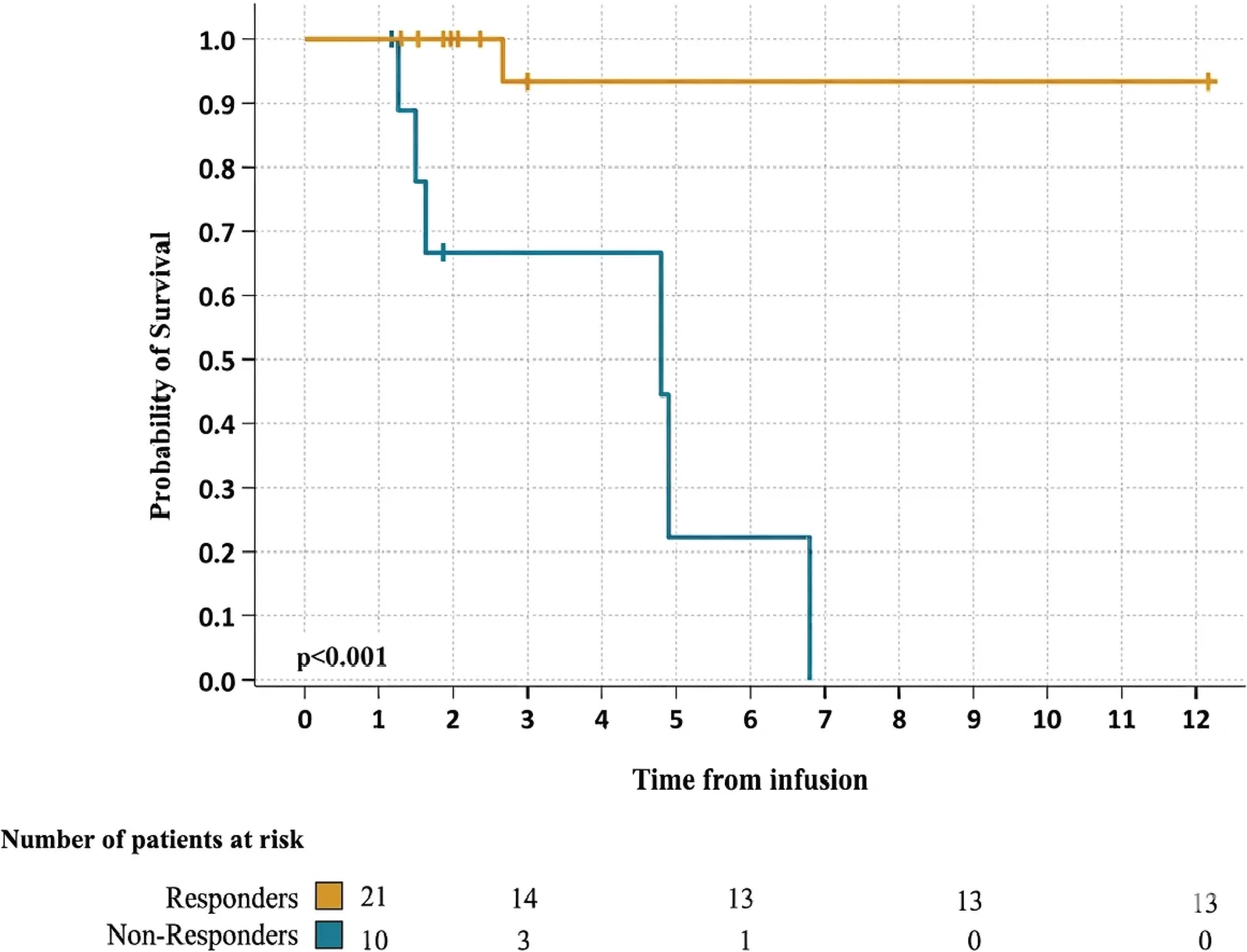
Landmark analysis of OS according to the 28-day response. This analysis includes 31 patients who remained in the study 28 days after their first VST infusion (see main text). Among these, 21 achieved at least an OR (VR/CRnesq/CR) by day 28 (gold line) and demonstrated significantly superior 1-year overall survival (93.3%, CI = 70.4–98.8) compared with the 10 nonresponders (blue line), who had a 1-year OS of 0%, (*P* < .001). The median survival time for nonresponders was 4.8 months (CI = 0.0–10.6). Among responders, the only death observed was due to leukemia progression. Crossed bars represent censored data. Kaplan–Meier estimates of overall survival were compared using the Log Rank test. The hazard ratio is 0.037, with a 95% CI of (0.004, 0.337). Abbreviations: CR, complete response; CRnesq, complete virologic response with persistent neurologic sequelae; OR, overall response; VR, virologic response.

### Reduction in CNS Viral Load Following Infusion of BK-VSTs

CSF JCV viral load was monitored by qPCR. Responders showed a significant decrease in viral load after BK-VST infusion, while nonresponders had stable or rising levels (Figure 3). Median PCR at day 28 was 1400 (0–12 000) for nonresponders, 0 (0–4200) for virologic responders, and 0 (0–0) for patients achieving CR/CRnesq. Among 16 patients with CR/CRnesq, median time to JCV clearance was 56 days (range 14–270 days; IQR 7.5).

**Figure 3.**
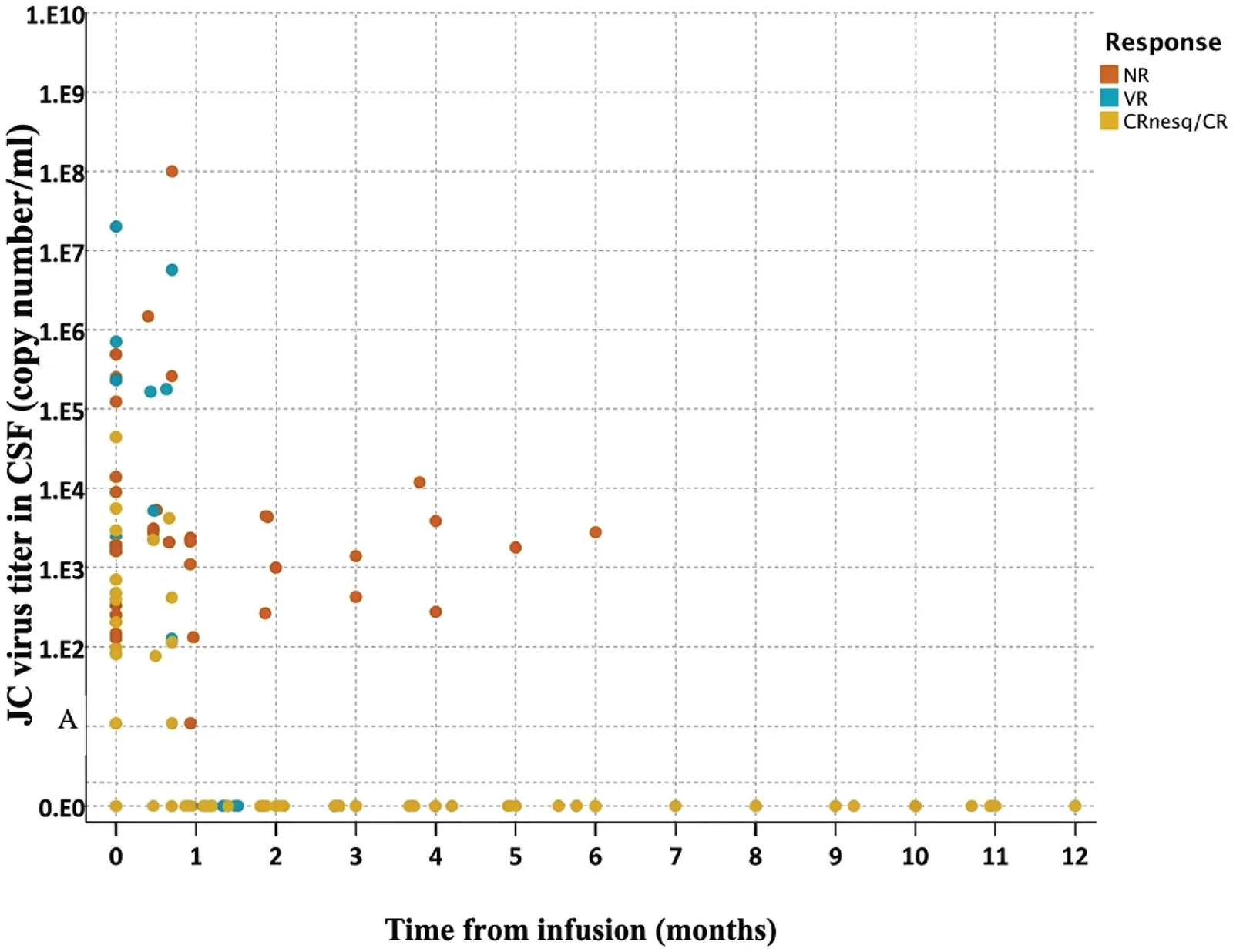
JC virus copy number in the CSF. The figure shows the evolution of JCV viral load over time. Patients were categorized based on their best response during follow-up: 16 patients achieved a CR or a CRnesq with neurological sequelae (gold), 5 patients showed a virological response without a clinical response (blue), and 16 patients failed to respond (brown). The viral load rapidly declined in responding patients but remained unchanged in nonresponders. The “A” on the Y axis represents patients with detectable but not quantifiable copy numbers, corresponding to a viral load of <72 copies/ml. The median PCR after 28 days for NR, VR, and CRnesq/CR was 1400 (0–12 000), 0 (0–4200), and 0 (0–0) respectively. Abbreviations: CR, complete response; CRnesq, complete virologic response with persistent neurologic sequelae; CSF, cerebrospinal fluid; JCV, JC polyomavirus; NR, nonresponder; OR, overall response, VR, virologic response.

### Pretherapy Predictors of Outcome

Patients with baseline CSF viral load above the 75th percentile (8200) had a significantly lower likelihood of achieving CR/CRnesq (OR = 0.10, 95% CrI = 0.008–0.618, PBE = 0.01). Similarly, patients with hematological malignancies were less likely to respond (OR = 0.26, 95% CrI 0.061–1.000, PBE = 0.02, Table 1).

**Table 1.** Patient Characteristics and Outcomes

Variable	N (%)	1-y CR/CRnesq, (95% CrI)	1-y OS
			HR, (95% CrI)
Age			
Median (IQR)	61 (19.5)	PBE = 0.45	PBE = 0.20
≤60 y	18 (48.6)	1	1
>60 y	19 (51.4)	0.93 (0.26–3.19)	1.39 (0.64–2.98)
Sex	…	PBE = 0.71	PBE = 0.44
Female	11 (29.7)	1	1
Male	26 (70.3)	1.48 (0.38–6.31)	1.08 (0.48–2.62)
Recipient of allogeneic SCT	…	PBE = 0.29	PBE = 0.16
No	33 (89.2)	1	1
Yes	4 (10.8)	0.68 (0.16–2.65)	2.85 (0.51–34.48)
Underlying disease	…	PBE = 0.02	PBE = 0.10
Other diagnosis^a^	14 (37.8)	1	1
Hematological malignancy^b^	23 (62.2)	0.26 (0.061–1.0)	1.65 (0.77–3.66)
Modified Rankin score^c^	…	PBE = 0.06	PBE = 0.14
0–1	0 (0.0)	-	-
2–3	8 (21.6)	1	1
4–5	22 (59.5)	0.25 (0.036–1.35)	1.64 (0.70–4.33)
CD4+ T-cell count in peripheral blood^d^			
Median (IQR) cells/µL	185 (294.5)	PBE = 0.55	PBE = 0.28
≤200 cells/µL	16 (43.2)	1	1
>200 cells/µL	13 (35.1)	1.09 (0.27–4.46)	1.29 (0.51–3.11)
CSF viral load			
Median (IQR)	1600 (11 386.5)	PBE = 0.01	PBE = 0.19
≤8200 copies/mL^e^	27 (73.0)	1	1
>8200 copies/mL	10 (27.0)	0.10 (0.008–0.62)	1.54 (0.516–4.01)
HLA match	…	PBE = 0.98	PBE = 0.99
Median (IQR)	3(2)	1.80 (1.006–3.48)	0.67 (0.46–0.95)

Due to the relatively small patient sample size, we examined the influence of the clinical variables in this table on outcomes using Bayesian models. In the Bayesian model, the influence of a given variable on outcome is quantified by the probability of a beneficial effect (PBE). Briefly, PBE is defined as the probability of a better outcome when the variable is present. A PBE near 0 indicates a harmful effect of the covariate, a PBE near 1 implies a beneficial effect, and a PBE around 0.50 corresponds to no significant effect.

Abbreviations: CR, complete response; CRnesq, complete virologic response with persistent neurologic sequelae; CSF, cerebrospinal fluid; IQR, interquartile range.

^a^Three patients were recipient of solid organ transplant (1 heart transplant, 1 renal transplant, and 1 lung transplant); 3 patients had immunodeficiency secondary to DiGeorge syndrome, idiopathic chronic pancytopenia, or idiopathic CD4 T-cell lymphopenia; 3 patients had autoimmune diseases (sarcoidosis, systemic lupus, and autoimmune hepatitis with autoimmune cytopenia); 4 patients had AIDS, 1 patient had a solid tumor.

^b^Six patients had myeloid malignancies and 17 patients had lymphoid malignances. Two patients with hematologic malignancies were recipients of CAR T cells (one following CAR19 and the other after BCMA CAR T cells).

^c^Seven patients had missing data.

^d^Eight patients had missing data.

^e^8200 representsthe 75th percentile.

Next, we investigated whether the degree of HLA matching between the patient and the VST influenced outcomes. The number of HLA matches (median 3, range 1–5) was statistically significantly associated to higher response rate (OR = 1.80, 95% CrI = 1.006–3.478, PBE = 0.98, Table 1) and superior overall survival (HR = 0.67, CrI = 0.46–0.95 PBE = 0.99).

### Patient Responses Were not Associated With Endogenous Immune Reconstitution

No evidence of spontaneous CD4+ T-cell recovery was observed during follow-up. In responders, the median CD4+ T-cell count was 190 cells// at baseline (range 22–563; IQR 201) and 258 cells// at response (range 38–951; IQR 338; *P* = .43, Wilcoxon–Mann–Whitney test).LL

In a subset with available samples (n = 13), peripheral blood CD45-positive cells were profiled by CyTOF at on the day of VST infusion (3 hrs post-infusion) and days 14 and 28 postinfusion. Persistence of donor-derived T cells was similar between patients with CR and NR (Supplementary Figure 4B) and did not differ by diagnostic group or degree of donor-recipient HLA matching (Supplementary Figure 4C and D). No significant differences were seen in CD19+ B cells, CD4+ T cells or CD8+ T cells between responders and nonresponders (Figure 4A–E), suggesting clinical responses were not due to endogenous immune reconstitution.

**Figure 4.**
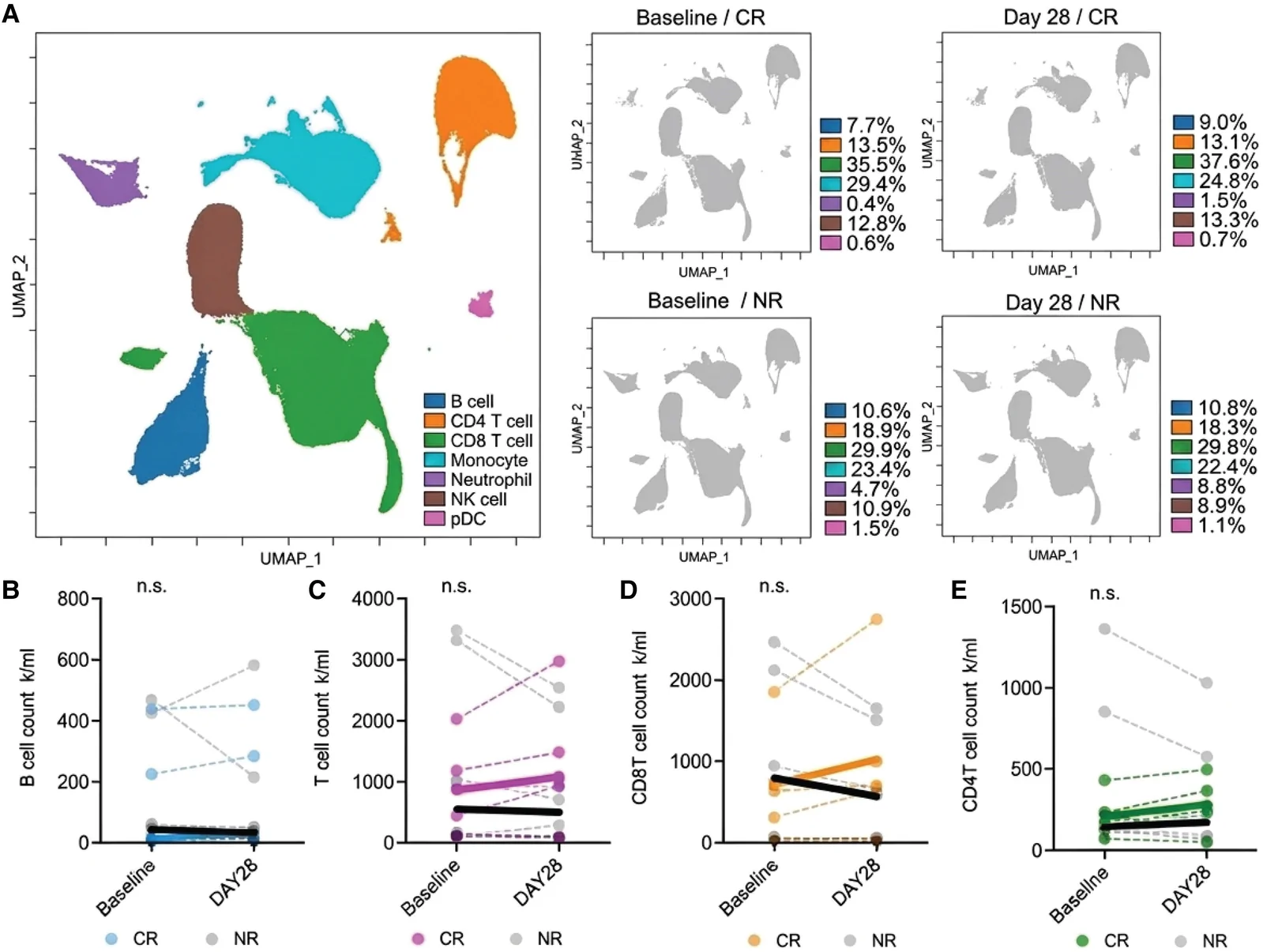
Phenotypic analysis of recipient CD45^+^ PBMCs preinfusion and postinfusion by mass cytometry. *A*, UMAP showing 7 major PBMC clusters derived from CyTOF analysis of peripheral blood samples from complete responders (CR/CRnesq, n = 5) and nonresponders (NRs, n = 8), merged into a single map. Each cluster is represented by a distinct color. Paired UMAPs display samples from baseline and Day 28 postinfusion for CR/CRnesq (top) and NRs (bottom). Frequencies of each cluster are shown next to each UMAP. *B*–*E*, Quantification of endogenous immune reconstitution, including (*B*) B cells, (*C*) total T cells, (*D*) CD8^+^ T cells, and (*E*) CD4^+^ T cells from PBMCs at baseline and Day 28 for CR/CRnesq (n = 5) and NRs (n = 8). Each dot represents an individual patient at a given time point; dashed lines connect paired samples from the same patient. Solid lines denote group medians. *P* values were calculated using a mixed-effects model with Geisser–Greenhouse correction.

### Donor T-cell Phenotype and Response

In 13 patients (5 CRs and 8 NRs) with informative donor-recipient HLA mismatches, CyTOF-based chimerism assays were used to determine the persistence and phenotype of donor-derived VSTs postinfusion. Identification of infused versus endogenous T cells required an informative HLA mismatch between donor and recipient, detectable by available antibody panels (Supplementary Figure 4A). Due to the limited availability of monoclonal antibodies against HLA markers (eg HLA-A2, -A3, -B6, -B7, -BW4), phenotyping was feasible only in a subset of patients. PBMCs were collected on Day 0 and at 28 days postinfusion. Uniform manifold approximation and projection (UMAP) analysis revealed 3 donor-derived T-cell clusters based on expression of CD45RA, CD45RO, CD27, CD28, CXCR3, EOMES, and T-BET. Cluster 2 cells were more abundant in responders, while cluster 3 predominated in nonresponders. Cluster 2 was enriched in CD4+ T cells expressing activation markers (CD25, CD69, CD95, and ICOS), transcription factors important for T-cell function (EOMES and T-BET), and chemokine receptors important for CNS trafficking (CCR5 and CXCR3) [32, 33] (Supplementary Figure 5A–C). In contrast, cluster 3 cells exhibited higher terminal differentiation and exhaustion markers (CD57, CD160, KLRG1, and TIGIT) and reduced homing receptors, suggesting an exhausted phenotype with diminished CNS trafficking capacity.

### Toxicity

BK-VSTs were well-tolerated, with no infusion-related reactions, pancytopenia, graft failure or GVHD observed. One patient developed clinical IRIS, defined as transient neurologic worsening accompanied by supportive MRI findings, including seizure-like activity, which resolved spontaneously with supportive care and without steroids. Three additional patients developed radiographic changes consistent with an inflammatory response, with MRI findings suggestive of immune-mediated inflammation but no corresponding clinical deterioration. No other toxicities were deemed related to VSTs. A comprehensive list of adverse events, regardless of attribution, is provided in Supplementary Table 6.

## DISCUSSION

We present results from a prospective study of adoptive T-cell therapy targeting JCV in PML. Treatment with partially HLA-matched, off-the-shelf BK virus-specific T cells resulted in a 56.8% OR, including 43.2% CR/CRnesq. Responses typically occurred within 28 days and were durable, with no loss of response during follow-up. Patients who responded by day +28 had markedly superior OS compared with nonresponders (93.3% vs 0%, *P* < .001).

Importantly, responses were observed regardless of underlying disease groups, including in patients with active or prior malignancy, a population historically associated with particularly poor prognosis [5, 10, 34, 35]. Lack of response in some patients may reflect host and disease-related factors, including the intensity of immunosuppression, disease burden, impaired T cell trafficking, altered antigen presentation or insufficient in vivo expansion of infused cells. In our cohort, 24 of the 37 patients had an underlying malignancy. This is an important distinction because although PML-associated malignancy is uncommon, accounting for only approximately 10% of PML cases globally [10], it is associated with >90% mortality within 2 months of diagnosis [5, 10, 34, 35]. To our knowledge, this study represents the largest prospective series evaluating adoptive T-cell therapy in this particularly vulnerable population.

Neurocognitive testing did not reveal significant group-level change from baseline to follow-up. However, RCI indicated individual cognitive improvement in most patients. Baseline cognitive function varied widely, likely reflecting PML heterogeneity. While some cognitive recovery was observed, most patients continued to experience deficits, highlighting that viral control does not ensure full neurological recovery.

MRI findings were concordant with clinical responses. Responders showed improvement in FLAIR lesions and radiographic features consistent with an inflammatory response, whereas nonresponders exhibited progressive lesion enlargement and more frequent diffusion restriction at lesion margins, consistent with active JCV.

Restoration of JCV-specific immunity is potentially curative, but endogenous immune reconstitution is often too slow to counter rapidly progressive PML. Adoptive VST therapy offers immediate delivery of anti-JCV immunity. In our study, most patients responded within 1 month of infusion. This rapid improvement, without changes in recipient T-cell counts or immune markers, supports the conclusion that infused VSTs were the primary driver of response. In a subset of patients with informative HLA mismatches, donor-derived VSTs were detected in blood using HLA-flow chimerism. We previously demonstrated that infused donor BK-VSTs home to recipient CSF and persist for several months. In this study, donor T cells exhibited effector phenotypes and expressed CNS-homing chemokine receptors (CCR5 and CXCR3), that was more abundant in responders.

VST therapy was well-tolerated, with no infusion-related reactions, pancytopenia, or GVHD despite HLA mismatches between donor VST products and recipients. One patient experienced clinical IRIS, which resolved without steroids or additional immunosuppression.

PML remains a devastating disease, and early diagnosis and treatment are critical. While autologous or bespoke allogeneic VSTs have shown promise [7], their production time can delay treatment and worsen outcomes. Our use of pre-established, HLA-diverse VST biobank enabled immediate treatment and offers a scalable model for clinical application. Although multivirus-specific T cells have been explored [21], we previously showed that dominant specificities (eg CMV) can outcompete BKV-specific responses, reducing potency and supporting the use of single-VST for BKV [23].

Immune checkpoint inhibitors, such as anti-PD-1 have also been used in PML but clinical responses have been variable and appear to depend at least in part on the presence of preexisting functional VSTs, which may be absent or profoundly impaired in severely immunocompromised patients [12, 13, 36–38]. Pembrolizumab was included because PD-1 blockade has been proposed as a strategy to restore or augment antiviral T-cell function in PML, particularly in patients with impaired cellular immunity. Since VSTs provide exogenous antiviral T cells, the rationale for the combination was that PD-1 blockade might enhance the activity, persistence or in vivo expansion of infused VSTs or improve endogenous antiviral immune responses. In our study, combining pembrolizumab with BK-VSTs was safe and feasible. Response rates were similar in patients who received pembrolizumab plus VSTs and those who received VSTs without pembrolizumab (Supplementary Table 5). Although our study was not designed to formally evaluate the additive clinical benefit of pembrolizumab, these data support BK-VSTs as the principal therapeutic strategy, with pembrolizumab considered selectively rather than as a necessary component of treatment.

Donor heterogeneity has been shown to influence response in both autologous and allogeneic cell therapy [25, 39]. In our trial, both patient and VST characteristics significantly impacted response. Low baseline CSF JCV load and the degree of HLA match between the VST lines and the patient emerged as the strongest predictors of achieving CR/CRnesq. The degree of HLA match was the only predictor for survival postinfusion.

Several limitations must be acknowledged. The small sample size limits precision of effect estimates, as reflected by wide credible intervals (Table 1). The predefined 1-year follow-up restricts long-term assessments and incomplete follow-up for some patients may further limit outcome interpretation. Multivariable modeling was not feasible and hazard ratios should be interpreted cautiously given their dependence on follow-up time and event distribution [40].

In conclusion, our findings highlight the potential of allogeneic BK-VSTs as an effective and well-tolerated therapy for PML. Responses were seen across diverse patient populations, including those with active malignancy, and were supported by improvements in clinical, virologic, radiographic, and survival outcomes. The ability to rapidly deliver off-the-shelf VSTs, combined with stringent donor selection, represents a significant therapeutic advance. Continued research is needed to validate these findings and establish BK-VSTs as a standard-of-care treatment for PML.

## Supplementary Material

ciag404_Supplementary_Data

## Supplementary Data

Supplementary materials are available at *Clinical Infectious Diseases* online. Consisting of data provided by the authors to benefit the reader, the posted materials are not copyedited and are the sole responsibility of the authors, so questions or comments should be addressed to the corresponding author.
