# Supplementary material for: Treatment of Progressive Multifocal Leukoencephalopathy With Third-Party Allogeneic BK Virus T Cells
Source: Clin Infect Dis. 2026 Jul 6;83(4):e777–86. doi: 10.1093/cid/ciag404 (PMC13715966; doi:10.1093/cid/ciag404)
Supplement: Supplementary file 1 [file ciag404_supplementary_data.pdf]

Supplementary Methods

Clinical Trial Design

Cerebrospinal fluid (CSF) was examined for JCV load before each infusion. Patients underwent serial clinical, neuropsychiatric, radiographic imaging, and CSF analyses. Patients with less than a complete response (CR) after the first infusion were eligible for additional infusions.

Patients

Immunosuppression was attributed to the following conditions: AIDS (n=4), hematologic malignancies (n=23), including 10 post-hematopoietic stem cell transplant (4 allogeneic, 6 autologous) and prior CAR T-cell therapy (n=2, one after CAR19 T-cells and the other after BCMA CAR T-cells). All patients with AIDS initiated anti-retroviral treatment at least 30 days prior to BK-VST infusion (median 2 months, range 1 month to 1 year). Additional causes of immunosuppression included solid organ transplantation (n=3; heart, lung and kidney), solid tumors (n=1), and primary or secondary immunodeficiencies (n=3; DiGeorge syndrome, idiopathic chronic pancytopenia, and idiopathic CD4 T-cell lymphopenia). Three patients had autoimmune diseases (sarcoidosis, systemic lupus erythematosus, autoimmune hepatitis with cytopenia), all of whom remained on steroid therapy (at a dose of less than 0.5mg/kg) after diagnosis of PML and during VST therapy.

Generation of BK-VSTs

Cells were expanded over 14 days in cytokine-supplemented media and tested for sterility, viability, and specificity prior to cryopreservation. Cells were not manipulated post cryopreservation but underwent QA testing prior to infusion. Functional activity was assessed by intracellular cytokine staining in response to polyomavirus overlapping peptides (pepmix). Cells were stimulated with pepmix from the 5 immunodominant polyomavirus proteins: small T, large T, VP1, VP2, VP3 (1 µg/mL each, JPT; Germany) in RPMI (Cytiva, Marlborough, MA) for 2 hours and resuspended at 1 x 10^6 /mL in RPMI media (Cytiva, Marlborough, MA) and Clicks media (Irvine Scientific, Santa Ana, CA) with human AB serum (Gemini Bioproducts), interleukin-2 (IL-2) (20 IU/mL; Prometheus, San Diego, CA), IL-7 (10 ng/mL; R&D Systems, Minneapolis, MN), and IL-15 (10 ng/mL R&D Systems). Cytokines were replenished every 2-3 days and cells were expanded for 14 days. At the end of the culture, VSTs were harvested and tested for aerobic and anaerobic sterility, mycoplasma, viability, endotoxin, and cytokine secretion before cryopreservation. Cytokine secretion by the VSTs in response to the polyomavirus pepmix was measured by intracellular flow cytometry. Briefly, VSTs were stimulated with polyomavirus pepmix (1 µg/mL of each peptide) in the presence of Brefeldin A (BFA) (Sigma Aldrich, St. Louis, MO) for 6 hours in media containing cytokines. Phorbol 12-myristate 13-acetate (PMA) and ionomycin were used as positive controls. The VSTs were stained for surface markers CD3, CD4, CD8, CD56 and CD45, permeabilized and stained for IL-2 and IFN-γ (BD Biosciences, San Jose, CA). Cells were read on the Canto-II flow cytometer and analyzed using the DIVA software.

Characterization of BK-VSTs

T-cell responses to viral antigens were assessed by intracellular flow cytometry for IFN-γ and IL-2. At least 500,000 live events were acquired, and data were analyzed with FlowJo. Donor T cells expressing IFN-γ after stimulation with polyomavirus pepmixes averaged 13.41% (range: 1.30%-31.09%), with predominantly CD4+ T-cell responses (**Supplementary Figure 1A-D**).

Statistical Methods

To assess associations between baseline covariates and outcomes, each variable was analyzed individually using Bayesian models. A Bayesian logistic regression model estimated the probability of response, while a Bayesian Weibull regression model assessed OS. Due to the small sample size, multivariable analyses were not performed. All analyses were conducted using noninformative or weakly informative priors. Posterior probabilities were used to quantify the likelihood of benefit (PBE), defined as the probability that the covariate had a beneficial effect (e.g., improved response or prolonged survival). The cumulative incidence (subdistribution) function of CRnesq was estimated using response as the event of interest and death as the competitor.(1, 2) Due to the limited sample size, multivariable regression models were not fit. Each patient covariate X was considered individually, with its effect on the probability of response estimated by a Bayesian logistic regression model with linear term beta0+beta1*X and -its effect on OS time estimated by a Bayesian Weibull regression model(3) including the same linear term in its log hazard parameter, assuming weakly informative default priors for a and b. The prior distribution for the shape parameter alpha was Gamma(0.1, 0.1), and the prior distribution for the beta parameters were Normal(0, 10).(4, 5) These models were assumed as reasonably robust choices due to the inability to perform model criticism reliably with the small sample. For each fitted regression model with linear term beta0+beta1*X for covariate X, a 95% posterior credible interval (CrI) was computed for the covariate parameter beta1. Since X=1 corresponds to a larger response probability, the effects of each X on response was quantified by the posterior probability of a beneficial effect, PBE = Pr(b>0|data). For OS, PBE was defined as Pr(beta1 < 0 | data), corresponding to a lower hazard and longer OS. Values of PBE close to 1 correspond to a large beneficial effect, values -close to 0 indicate a small beneficial (large harmful) effect;, -and a PBE close to .50 corresponds to little or no effect.(3, 4) Each model was fit with a burn-in period of 1000 iterations followed by a total of 10,000 iterations with no thinning performed. Posteriors were computed using Markov chain Monte Carlo in JAGS version 4.3.2. Due to the small sample size of 37, the reliability of any inferences is limited and should not be overinterpreted.(1, 2, 5)

Supplementary Figures

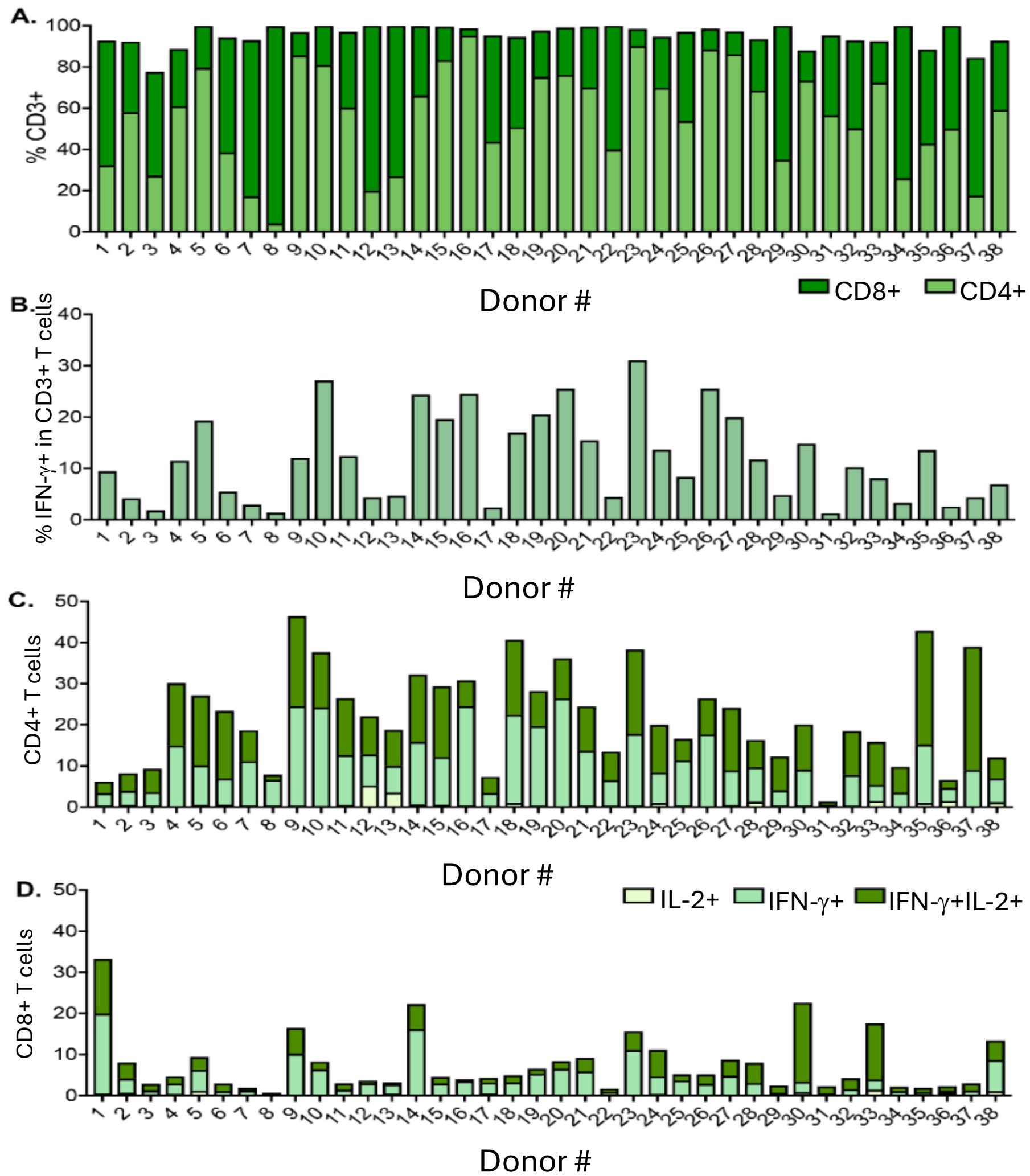

Supplementary Figure 1. Phenotype of polyomavirus-specific T cell clinical products. A. Bar plot showing the percentage (%) of $CD8^+$ T cells and $CD4^+$ T cells in total $CD3^+$ T cells. Each bar represents one donor (n=38). **B**. Bar plot showing the percentage (%) of T cells secreting IFN-γ after stimulation with polyomavirus-derived peptides. **C-D**. Bar plot showing the percentage (%) of CD4 T cells (**C**) or CD8 T cells (**D**) secreting IL-2 and/or IFN-γ after stimulation with polyomavirus-derived peptides (n=38).

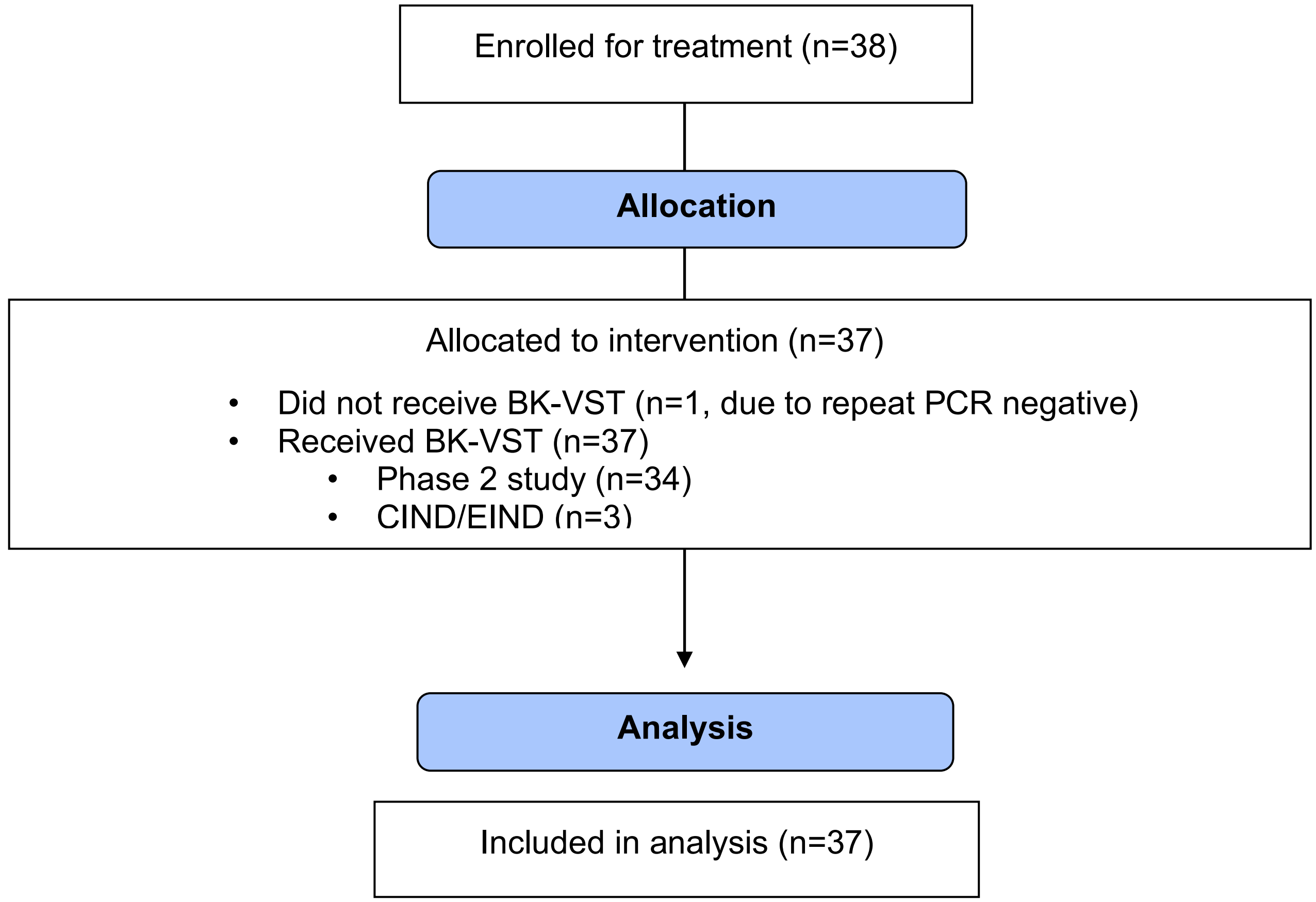

Supplementary Figure 2: Study Cohort Flow Diagram. Thirty-eight patients were enrolled for treatment. One patient did not receive BK virus-specific T cells (BK-VSTs) because repeat PCR testing was negative for JCV. A total of 37 patients received BK-VSTs and were included in the analysis, including 34 treated on the phase 2 study and 3 treated under compassionate or emergency IND mechanisms.

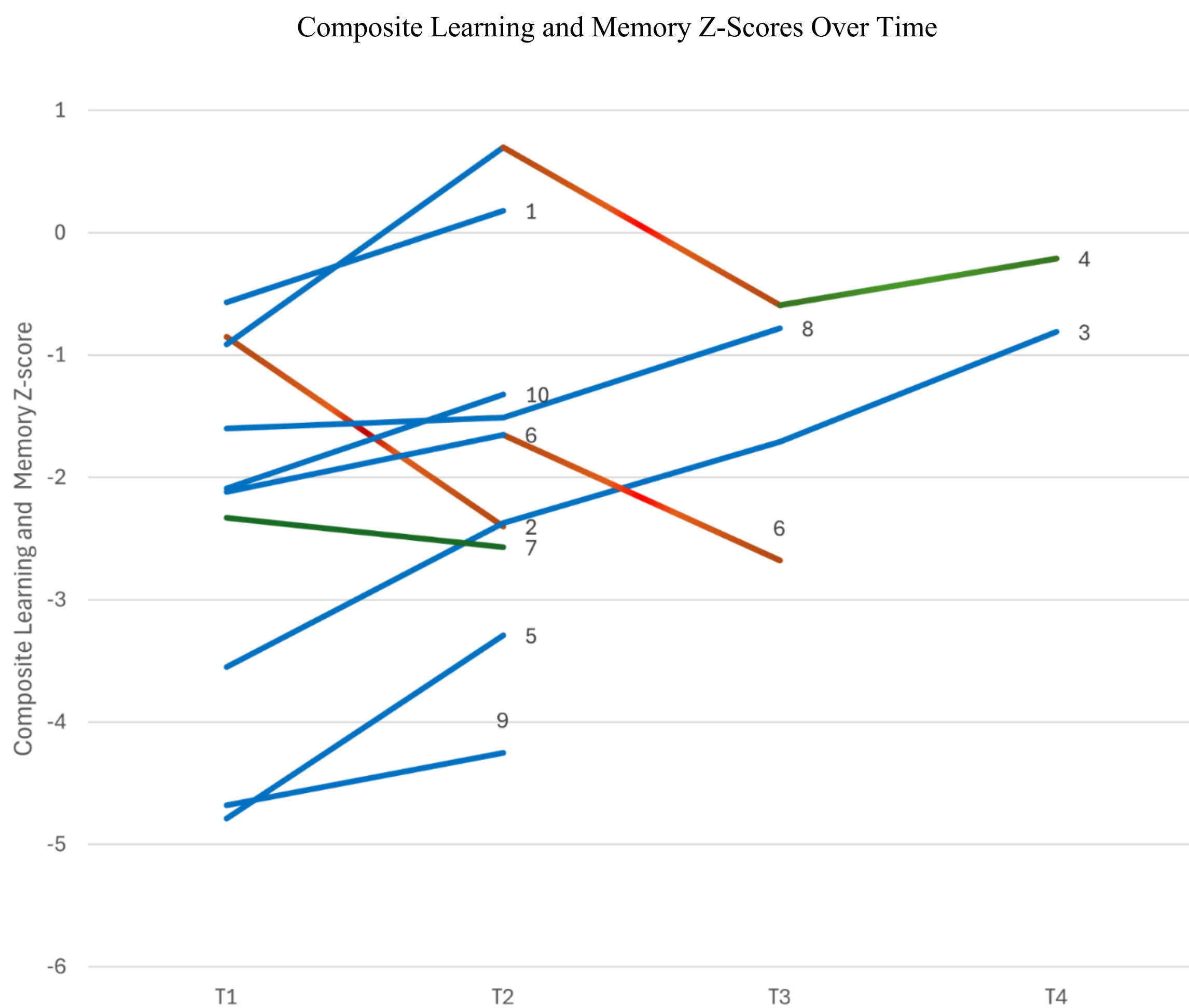

Supplementary Figure 3. Longitudinal trajectories of composite memory Z scores at four assessment timepoints (T1-T4). For each test interval, significant positive change (improved) is color coded blue, significant negative change (declined) is color coded red, no significant change is color coded green. Ten participants completed assessments at T1 and T2; four participants completed T3; two participants completed T4.

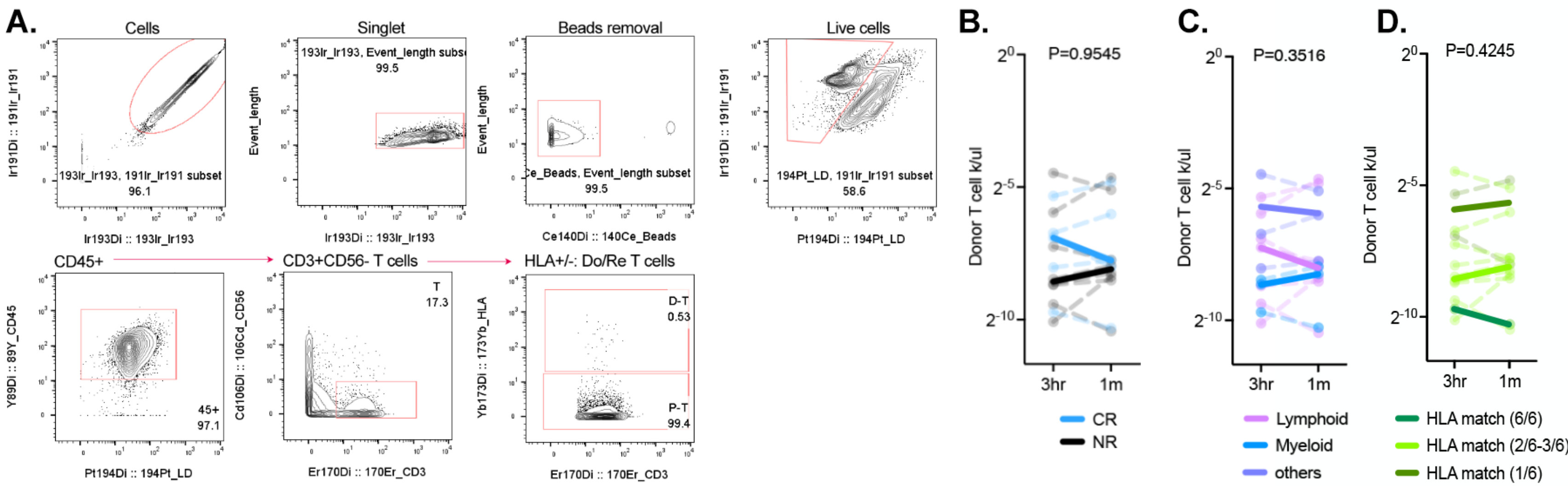

Supplementary Figure 4. Detection of donor-derived T cells after infusion in patients. **A**. Sequential gating strategy was used to determine donor-derived T cells. For this, cells were gated excluding debris, and then singlets were used to check the viability. CD45+ Live cells were further split on CD56 and CD3 axis, and T cell subsets were defined as CD56-/CD3+ cells. Then, whenever possible, donor specific HLA was used to distinguish donor T cell from patient. **B**. Dot plot showing the absolute cell count of donor-derived T cells from peripheral blood (PB) samples of responders (CRs; n=5), vs. non-responders (NRs; N=8) after 3hr or 28 days after treatment. Dot from the same patient was lined up. P value was determined using two-sided two-way ANOVA. **C**. Patients with lymphoid-derived disease (n=7), myeloid-derived disease (n=3) and other (n=3), and **D.** Persistence of infused VSTs according to donor recipient HLA match 1 month after infusion in patients receiving VST products with HLA matching of 6/6 (n=1), 2/6 and 3/6 (n=10), or 1/6 (n=2). Dot from the same patient was lined up. P value was determined using two-side two-way RM ANOVA.

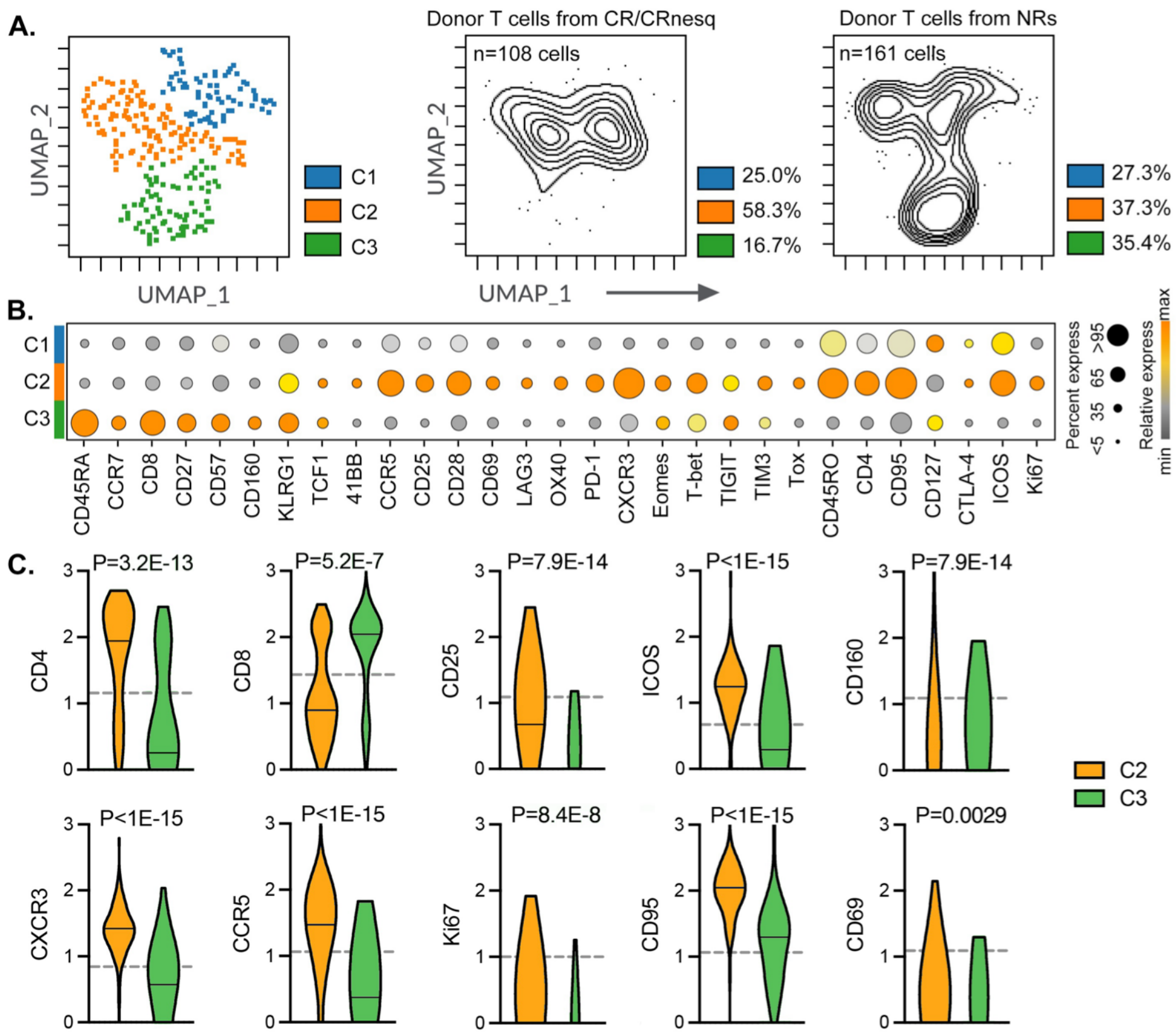

Supplementary Figure 5. Characterization of donor-derived T cells post-infusion using mass cytometry. A. UMAP analysis showing 3 main donor-derived T-cell clusters. Donor-specific T cells in the PB of patients with CR/CRnesq and non-responders (NRs) were identified based on distinct HLA mismatches (A2, A3, B6, B7, and BW4) using CyTOF. Cells were merged to create a single UMAP, with each cluster represented by a different color. The middle panel shows UMAP for T cells from responders, and the right panel for NRs, with cluster frequencies indicated next to each UMAP. **B.** Heatmap depicting the expression of key T-cell phenotypic and functional markers. Marker intensity is represented on a gray-to-orange color scale (low to high), while circle size indicates the percentage expression, with larger circles corresponding to higher percentages. **C.** Violin plots comparing the expression of individual markers between cells in cluster 2 (dominant in CR/CRnesq) and cells in cluster 3 (dominant in NRs). Statistical significance was assessed using a two-sided Wilcoxon-Mann-Whitney test.

Supplementary Table 1. Table of Assessments.

2014-0279<br>JC Virus Assessment Table	Within 2 weeks of enrollment (Consent Date)	Within 72 hours of BK-VST infusion	BK- VST infusion					
	Pre-BK-VST Infusion		Day 0	Once between Week 4 and Week 8 (+/- 2 days)	3 Months	6 Months	9 Months	12 Months
Complete History and Physical	X							
Follow up Note and Physical		X[6]		X[3]	X	X		X
Weight (weekly until Day 45)	X			X[3]				
Neurocognitive consult	X[1]				X[1]	X[1]		
Neurocognitive consult (EDSS scoring)	X[1]				X[1]	X[1]		
CBC w/ diff, platelets[4]	X[2]			X[3]	X	X		X
Electrolytes (Na, Cl, K, CO2), glucose[5]	X[2]			X[3]	X	X		X
Renal Function Tests (BUN, Creatinine)[5]	X[2]			X[3]	X	X		X
LFT (Tot. bili, SGPT/SGOT, alk phos, LDH,<br>Albumin, Total Protein[5]	X			X[3]	X	X		X
HLA Typing (Autologous and non Stem Cell Transplant patients only)	X[4]							
**JC Viremia**				X	X	X	X	X
BHCG serum pregnancy test	X							
Chest X-ray	X	X						
JCV Plasma PCR, JCV CSF by PCR, myelin<br>basic protein, CD4 panel, CD8 panel, cytology (lumbar puncture)	X			X[3]	X	X		X
MRI Brain[5]	X			X[3]	X	X		X
Adverse Events assessment/grading				X	X	X		X
Acute GVHD Staging[6]				X[3]				
Chronic GVHD Evaluation[6]					X			X
Total steroid dose				X[3]	X	X	X	
Viral Response determination				X				
Secondary graft failure determination (stem<br>cell transplant recipients only)				X				
Correlative Studies (required):								
Phenotype			X[7]	X	X	X		X
VST precursor frequency			X[7]	X	X	X		X

[1] For JCV patients with encephalitis: Neurological consult and full exam with EDSS score within one week of enrollment, at 3 months (+/- 14 days) and 6 months (+/- 28 days) post each VST infusion, and as clinically indicated.

[2] Baseline laboratory evaluations: Hematology (CBC w/diff and platelets), complete liver enzymes testing (total bilirubin, SGPT or SGOT, alkaline myelin basic protein from CSF, CD4 panel, CD8 panel, JCV plasma by PCR and JCV CSF by PCR. MRI of brain. Serum pregnancy test if participant has childbearing potential.

[3] Once between Week 4 and Week 8 (+/- 2 days) and at the discretion of the PI.

[4] Any time before enrollment.

[5] Starting 4 weeks post VST infusion: Laboratory tests and MRI scans can be performed locally and results sent to MDACC. The PI/treating physician must review outside lab and MRI results, determine clinical significance, sign and date the outside lab and MRI results.

[6] Acute GVHD staging and grading information including assessments of rash, diarrhea, N/V, weight and liver function tests (LFT).

[7] Day 0 (- 2 days)

Supplementary Table 2A. List of CyTOF antibodies used for T cell phenotyping.

Antibodies	Clone	Source	Cat #	Antibody Dilutions
CD45	HI30	Standard Biotools	3089003B	1:167
CD56	REA196	Miltenyi Biotec	130-108-016	1:167
CD19	REA675	Miltenyi Biotec	130-122-301	1:250
CD14	REA599	Miltenyi Biotec	130-122-290	1:250
CD4	REA623	Miltenyi Biotec	130-122-238	1:250
CD8	REA734	Miltenyi Biotec	130-122-281	1:250
CD27	REA499	Miltenyi Biotec	130-122-295	1:250
CD45RA	REA562	Miltenyi Biotec	130-122-292	1:167
CD57	REA769	Miltenyi Biotec	130-124-525	1:500
CD45RO	REA611	Miltenyi Biotec	130-124-323	1:167
CCR6	REA190	Miltenyi Biotec	130-108-023	1:125
EOMES	WD1928	Invitrogen	14-4877-82	1:167
CD127	A019D5	Standard Biotools	3143012B	1:200
CCR5	REA245	Miltenyi Biotec	130-122-313	1:125
TCRγδ	REA591	Miltenyi Biotec	130-122-291	1:250
LAG3	REA351	Miltenyi Biotec	130-124-529	1:167
CCR4	REA279	Miltenyi Biotec	130-122-323	1:125
CD28	REA612	Miltenyi Biotec	130-122-350	1:167
CD25	REA570	Miltenyi Biotec	130-122-302	1:334
CD69	REA824	Miltenyi Biotec	130-124-326	1:5000
2B4	REA112	Miltenyi Biotec	130-124-523	1:500
TNF-α	Mab11	Standard Biotools	3152002B	1:167
TIM3	REA635	Miltenyi Biotec	130-122-333	1:125
CTLA-4	REA1003	Miltenyi Biotec	Custom order	1:125
TOX	REA473	Miltenyi Biotec	130-126-455	1:100
CXCR3	REA232	Miltenyi Biotec	130-108-022	1:167

**Antibodies**	**Clone**	**Source**	**Cat #**	**Antibody Dilutions**
OX40	ACT35	Standard Biotools	3158012B	1:125
CD160	BY55	Biolegend	341202	1:167
T-BET	4B10	Standard Biotools	3160010B	1:250
TIGIT	REA1004	Miltenyi Biotec	130-122-310	1:125
Ki67	B56	Standard Biotools	3162012B	1:250
TCRv7a.2	REA179	Miltenyi Biotec	130-126-467	1:167
TCF1	7F11A10	Biolegend	655202	1:167
PD1	PD1.3.1.3	Miltenyi Biotec	130-096-168	1:125
IL2	MQ1-17H12	Standard Biotools	3166002B	1:125
CCR7	REA546	Miltenyi Biotec	130-122-300	1:167
IFN-γ	B27	Standard Biotools	3168005B	1:167
FOXP3	REA1253	Miltenyi Biotec	Custom order	1:125
CD3	UCHT1	Standard Biotools	3170001B	1:250
MIP1-β	24006	R&D Systems	MAB271	1:125
KLRG1	REA261	Miltenyi Biotec	130-126-458	1:125
HLA	REA145	Miltenyi Biotec	Custom order	1:125
CD107a	REA792	Miltenyi Biotec	130-124-536	1:500
CD95	DX2	Miltenyi Biotec	130-108-066	1:250
ICOS	REA192	Miltenyi Biotec	130-122-304	1:167
LD		Standard Biotools	201194	1:5000
CD39	MZ18-23C8	Miltenyi Biotec	130-093-506	1:125
Perforin	B-D48	Standard Biotools	3196002B	1:100
Granzyme B	GB11	Standard Biotools	3198002B	1:100
4-1BB	4B4-1	Standard Biotools	3209015B	1:125

Supplementary Table 2B. List of CyTOF antibodies used for immune profiling.

Antibodies	Clone	Source	Cat #	Antibody Dilutions
CD45	HI30	Standard Biotools	3089003B	1:167
CD56	REA196	Miltenyi Biotec	130-108-016	1:167
CD19	REA675	Miltenyi Biotec	130-122-301	1:250
CD4	REA623	Miltenyi Biotec	130-122-238	1:250
CD8	REA734	Miltenyi Biotec	130-122-281	1:250
CD15	HI98	BD Pharminogen	555400	1:500
CD14	63D3	Biolegend	367102	1:167
CD57	REA769	Miltenyi Biotec	130-124-525	1:500
CD1c	AD5-8E7	Miltenyi Biotec	130-090-695	1:100
CCR6	REA190	Miltenyi Biotec	130-108-023	1:125
CD36	REA760	Miltenyi Biotec	130-124-322	1:334
CD127	A019D5	Standard Biotools	3143012B	1:200
CCR5	REA245	Miltenyi Biotec	130-122-313	1:125
IgD	REA740	Miltenyi Biotec	130-124-308	1:167
LAG3	REA351	Miltenyi Biotec	130-124-529	1:167
CCR2	REA264	Miltenyi Biotec	130-122-319	1:125
CD64	REA978	Miltenyi Biotec	130-124-325	1:250
CD25	REA570	Miltenyi Biotec	130-122-302	1:334
CD86	REA968	Miltenyi Biotec	130-122-334	1:125
CD123	REA918	Miltenyi Biotec	130-122-297	1:250
TCRγδ	REA591	Miltenyi Biotec	130-122-291	1:250
TIM3	F38-2E2	Standard Biotools	3153008B	1:125
CX3CR1	REA385	Miltenyi Biotec	130-122-286	1:125
CD27	REA499	Miltenyi Biotec	130-122-295	1:250
CXCR3	G025H7	Standard Biotools	3156004B	1:167
CCR4	REA279	Miltenyi Biotec	130-122-323	1:125
CD11c	Bu15	Standard Biotools	3159001B	1:250
PD1	PD1.3.1.3	Miltenyi Biotec	130-096-168	1:125

**Antibodies**	**Clone**	**Source**	**Cat #**	**Antibody Dilutions**
TIGIT	REA1004	Miltenyi Biotec	130-122-310	1:125
CD66B	80H3	Standard Biotools	3162023B	1:250
TCR Vα7.2	REA179	Miltenyi Biotec	130-126-467	1:167
CD45RO	REA611	Miltenyi Biotec	130-124-323	1:167
CD163	REA812	Miltenyi Biotec	130-122-293	1:167
CD24	REA832	Miltenyi Biotec	130-124-316	1:100
CCR7	REA546	Miltenyi Biotec	130-122-300	1:167
CD206	15-2	Standard Biotools	3168008B	1:100
CD45RA	REA562	Miltenyi Biotec	130-122-292	1:167
CD3	UCHT1	Standard Biotools	3170001B	1:250
CXCR5	REA103	Miltenyi Biotec	130-122-325	1:334
KLRG1	REA261	Miltenyi Biotec	130-126-458	1:125
HLA	REA145	Miltenyi Biotec	130-095-212	1:125
CD161	191B8	Miltenyi Biotec	130-092-676	1:125
CD95	DX2	Miltenyi Biotec	130-108-066	1:250
PD-L1	MIH2	Miltenyi Biotec	393602	1:167
LD		Standard Biotools	201194	1:5000
CD39	MZ18-23C8	Miltenyi Biotec	130-093-506	1:125
HLA-DR	REA805	Miltenyi Biotec	130-122-299	1:250
CD294	BM16	Biolegend	350102	1:167
CD16	3G8	Standard Biotools	3209002B	1:167

Supplementary Table 3. The patient's score on the Modified Rankin Scale (mRS) at the time of study enrollment prior to receiving BK virus-specific T cells and after therapy.

mRS Baseline	mRS After Therapy	Response
4	0	CR
4	3	CRnesq
4	4	NR
4	6	NR
4	2	CRnesq
5	0	CR
5	5	NR
4	4	NR
4	4	NR
4	4	NR
2	0	CR
2	1	CRnesq
4	4	NR
4	3	CRnesq
3	6	NR
3	1	CRnesq
4	4	NR
4	5	NR
4	0	CR
4	2	CRnesq
3	3	VR
4	4	VR
N/A	N/A	NR
N/A	N/A	NR
4	N/A	VR
4	1	CRnesq

3	2	CRnesq
4	3	CRnesq
3	2	CRnesq
3	3	CRnesq
N/A	N/A	NR
N/A	N/A	NR
N/A	N/A	NR
N/A	N/A	VR
4	4	VR
4	6	NR
N/A	N/A	CR

CR: Complete Response, CRnesq: Complete Response with neurologic sequelae, VR: virologic response; NR: non-response, N/A: Not available,

Supplementary Table 4. Reliable Change Index Individual-level neurocognitive performance across timepoints (T1-T4).

Patient	Test	T1 Raw	T1 Z	T1 Class	T1 Comp Z	T2 Raw	T2 Z	T2 Class	T2 Comp Z	RCI T2-T1 Raw	RCI T2-T1	T3 Raw	T3 Z	T3 Class	T3 Comp Z	RCI T3-T2 Raw	RCI T3-T2	T4 Raw	T4 Z	T4 Class	T4 Comp Z	RCI T4-T3 Raw	RCI T4-T3	RCI Final Raw	RCI Final
1	HVLT Tot	23	-0.4	Avg	-0.57	26	0.15	Avg	0.18	3	NC													3	NC
	HVLT Del	8	-0.25	Avg		9	0.11	Avg		1	NC													1	NC
	HVLT Rec	9	-1.07	Lo Avg		11	0.27	Avg		2	Improved													2	Improved
2	HVLT Tot	25	-0.58	Avg	-0.85	22	-1.28	Lo Avg	-2.4	-3	NC													-3	NC
	HVLT Del	6	-2.11	Mod imp		4	-3.22	Severe imp		-2	NC													-2	NC
	HVLT Rec	11	0.14	Avg		7	-2.71	Mod imp		-4	Declined													-4	Declined
3	HVLT Tot	12	-3.6	Severe imp	-3.55	14	-3.14	Severe imp	-2.37	2	NC	20	-1.74	Mild imp	-1.71	6	Improved	20	-1.74	Mild imp	-0.81	0	NC	8	Improved
	HVLT Del	2	-4.33	Severe imp		5	-2.67	Mod imp		3	Improved	6	-2.11	Mod imp		1	NC	7	-1.56	Mild imp		1	NC	5	Improved
	HVLT Rec	7	-2.71	Mod imp		9	-1.29	Lo Avg		2	Improved	9	-1.29	Lo Avg		0	NC	12	0.86	Hi avg		3	Improved	5	Improved
4	HVLT Tot	27	-0.12	Avg	-0.91	30	0.58	Avg	0.7	3	NC	23	-1.05	Lo avg	-0.59	-7	Declined	23	-1.05	Lo avg	-0.21	0	NC	-4	NC
	HVLT Del	10	0.11	Avg		11	0.67	Hi avg		1	NC	9	-0.44	Avg		-2	NC	9	-0.44	Avg		0	NC	-1	NC
	HVLT Rec	7	-2.71	Mod imp		12	0.86	Hi avg		5	Improved	11	-0.27	Avg		-1	NC	12	0.86	Hi avg		1	NC	5	Improved
5	HVLT Tot	10	-4.07	Severe imp	-4.79	13	-3.37	Severe imp	-3.29	3	NC													3	NC
	HVLT Del	0	-5.44	Severe imp		3	-3.78	Severe imp		3	Improved													3	Improved
	HVLT Rec	4	-4.86	Severe imp		7	-2.71	Mod imp		3	Improved													3	Improved
6	HVLT Tot	16	-3.37	Severe imp	-2.12	18	-2.84	Mod imp	-1.65	2	NC	13	-4.16	Severe imp	-2.68	-5	Declined							-3	NC
	HVLT Del	4	-3.71	Severe imp		7	-1.94	Mild imp		3	Improved	5	-3.12	Severe imp		-2	NC							1	NC
	HVLT Rec	12	0.73	Hi avg		11	-0.18	Avg		-1	NC	11	-0.75	Lo avg		0	NC							-1	NC
7	HVLT Tot	16	-3.37	Severe imp	-2.33	19	-2.58	Mod imp	-2.57	3	NC													3	NC
	HVLT Del	6	-2.53	Mod imp		5	-3.12	Severe imp		-1	NC													-1	NC
	HVLT Rec	10	-1.09	Lo Avg		9	-2	Mod imp		-1	NC													-1	NC
8	HVLT Tot	21	-0.76	Lo Avg	-1.6	17	-1.49	Mild imp	-1.51	-4	NC	22	-0.58	Avg	-0.78	5	Improved							1	NC
	HVLT Del	6	-0.96	Lo Avg		5	-1.32	Mild imp		-1	NC	5	-1.32	Mild imp		0	NC							-1	NC
	HVLT Rec	6	-3.07	Severe imp		8	-1.73	Mild imp		2	Improved	10	-0.44	Avg		2	Improved							4	Improved

**9**	HVLT Tot	13	-4.16	Severe imp	-4.68	11	-4.68	Severe imp	-4.25	-2	NC			-2	NC
	HVLT Del	0	-6.06	Severe imp		0	-6.06	Severe imp		0	NC			0	NC
	HVLT Rec	7	-3.82	Severe imp		9	-2	Mod imp		2	Improved			2	Improved
**10**	BVMT Tot	5	-2.75	Mod imp	-2.09	19	-0.39	Avg	-1.32	14	Improved			14	Improved
	BVMT Del	3	-1.64	Mild imp		4	-1.7	Mild imp		1	NC			1	NC
	BVMT Rec	3	-1.88	Mild imp		3	-1.88	Mild imp		0	NC			0	NC

Raw scores, Z-scores, and clinical classifications are shown for each test and participant. Composite Z-scores represent aggregated performance across memory domains [average of z-scores of total learning (Tot) delayed recall (Del), and recognition (Rec)]. RCI = Reliable Change Index. The clinical classifications (class) describe performance within the range of standard deviations from the normative mean based upon comparison to age-appropriate statistical norms: Severely Impaired (-3.00 or less), Moderately Impaired (-2.99 to - 2.00), Mildly Impaired (-1.99 to -1.34), Low Average (-1.33 to -0.67), Average (-0.66 to 0.66), High Average (0.67 to 1.33), Superior (1.34 to 2.00), Very Superior (>2.00). *(Abbreviations: Avg: average, Lo Avg: low average, Mod imp: moderately impaired, Severe imp: severely impaired, NC: no change)*

HVLT Tot = verbal learning, HVLT Del = verbal delayed recall, HVLT Rec = verbal recognition, BVMT Tot = visual learning, BVMT Del = visual delayed recall, BVMT Rec = visual recognition

Demonstration on neurocognitive improvement (Improved), no change (NC), or significant decline (Decline) from the previous evaluation for each of the three aspects of memory (learning, delayed recall, recognition). Eight of 10 patients showed improvement based on the RCI on at least one aspect of memory compared to the previous evaluation.

Supplementary Table 5. Comparison of outcomes across three groups, the entire patient cohort, the nine patients who either received BK-VSTs plus pembrolizumab or experienced a prolonged clinical course, and the remaining 28 patients who were treated exclusively with BK-VSTs.

	1-month OR, n (%)	1-year CR/CRnesq, n (%)	1-year OS (95% CI)
**Whole cohort (n=37)**	21 (56)	16 (43)	61.1% (41- 77)
**Patients treated with VSTs plus pembrolizumab or with prolonged disease (n=9)**	5 (55)	3 (33)	50.8 (17-83)
**Patients treated exclusively with VSTs (n=28)**	16 (57)	13 (46)	63.9 (42-81)

Supplementary Table 6. Adverse events following BK-VST infusion. Abbreviations: UNR: Unrelated; UNL: Unlikely Related; PO: Possibly Related; PR: Probably Related. Based on NCI Common Terminology Criteria for Adverse Events (CTCAE), Version 4.0.(6)

Adverse Event	Grade 1	Grade 1 Attribution	Grade 2	Grade 2 Attribution	Grade 3	Grade 3 Attribution	Grade 4	Grade 4 Attribution	Grade 5	Grade 5 Attribution
**Cardiovascular**										
Sinus Tachycardia	3	UNR	1	UNL						
**Respiratory**										
Cough	1	1 UNR			2	1 UNR<br>1 PO				
Hypoxia	0		0		0					
**Gastrointestinal**										
Constipation	4	4 UNR	1	UNR						
Diarrhea	1									
Nausea	1	UNR	5	UNR	1	UNR				
Vomiting	2	1 UNR<br>1 PO	1	UNR	1	UNR				
Anorexia	1	UNR	2	2 UNR	1	UNR				
**Genitourinary**										
Acute Kidney Injury	0		0		0					
Urinary Urgency	0		1	UNR	0					
**Musculoskeletal**										
Muscle Weakness/Generalized	2	UNR	6	UNR	8	7 UNR<br>1 PO				
Muscle Weakness left sided	0		6	UNR	3	UNR				
Muscle Weakness right sided	2	UNR	7	UNR	4	UNR				
Facial Muscle Weakness	3		5	UNR						
Myalgia/Arthralgia	0		0		0					

Adverse Event	Grade 1	Grade 1 Attribution	Grade 2	Grade 2 Attribution	Grade 3	Grade 3 Attribution	Grade 4	Grade 4 Attribution	Grade 5	Grade 5 Attribution
**Ophthalmologic**										
Diplopia/Blurred Vision	0		5	UNR	0					
**ENT**										
Dysarthria	13	UNR	10	UNR	5	UNR				
Dysphagia	5	UNR	10	UNR	7	UNR				
Voice change	0		0		1	UNR				
Headache	5	UNR	0		2	UNR				
Nasal Congestion	2	UNR	0		0					
Mucositis	1	UNR	2	UNR	0					
**Skin**										
Skin Infection	0		0		1	UNR				
Dry Skin/Erythema/Pruritis	0		0		0					
Rash	3	1 UNR<br>2 PO	4	3 UNR<br>1 PO	1	PO				
**Neuropsychiatric**										
Agitation	1	UNR	2	UNR	0					
Confusion	3	UNR	3	UNR	3	UNR				
Memory Impairment	6	UNR	3	UNR	0					
Depression	4	UNR			1	PO				
Anxiety	3	UNR	2	UNR	0					
Dizziness	6	UNR	0		0					
Gait Disturbance	5	UNR	7	UNR	11	UNR				
Fall	5	UNR	0		1	UNR				
Ataxia	4	UNR	17	UNR	6	UNR				
Involuntary Movement	0		0		0					
Paresthesia	2	UNR	5	4 UNR<br>1 PO	3	UNR				

Adverse Event	Grade 1	Grade 1 Attribution	Grade 2	Grade 2 Attribution	Grade 3	Grade 3 Attribution	Grade 4	Grade 4 Attribution	Grade 5	Grade 5 Attribution
Seizure	3	2 UNR<br>1 PO	1	UNR	1	UNL	1	PR		
Somnolence	2	UNR	0		0					
Fatigue	5	UNR	12	UNR	8	7 UNR<br>1 PO				
Insomnia	1	UNR	1	UNR	0					
Spasticity	2	UNR	2	UNR	2	UNR				
Tremors	2	UNR	1	UNR	0					
**Infection**										
Encephalitis	0		0		0		0		1	UNR
Pneumonia	0		0		0					
Upper Respiratory Infection					1	UNR				
**Miscellaneous**										
Edema	2	UNR	0		0					
Injury	0		1	UNR	0					
Cold Intolerance	0		0		0					

Supplementary References

1. Kaplan EL, Meier P. Nonparametric Estimation from Incomplete Observations. Journal of the American Statistical Association. 1958;53(282):457–81.
2. Fine JP, Gray RJ. A Proportional Hazards Model for the Subdistribution of a Competing Risk. Journal of the American Statistical Association. 1999;94(446):496-509.
3. Gelman A, Carlin JB, Stern HS, Rubin DB. Bayesian Data Analysis. 2 ed. New York: Chapman and Hall/CRC; 2003.
4. Gelman A, Jakulin A, Pittau MG, Su Y. A weakly informative default prior distribution for logistic and other regression models. Ann Appl Stat. 2008;2(4):1360-83.
5. Hernán MA. The hazards of hazard ratios. Epidemiology. 2010;21(1):13-5.
6. Common Terminology Criteria for Adverse Events (CTCAE): National Cancer Institute; 2010 [Available from: https://ctep.cancer.gov/protocoldevelopment/electronic_applications/ctc.htm#ctc_40.
